# Detectable effects of bacterial phylogeny on virulence but not phage efficacy in an *in vivo Galleria mellonella* model

**DOI:** 10.1093/ismeco/ycag232

**Published:** 2026-08-20

**Authors:** Sarah K Walsh, Ryan M Imrie, Angus Buckling, Ben Longdon

**Affiliations:** Medical Research Council-University of Glasgow Centre for Virus Research, School of Infection and Immunity, University of Glasgow, Glasgow, Scotland, G611QH, United Kingdom; Centre for Ecology and Conservation; Faculty of Environment, Science, and Economy; Biosciences, University of Exeter; Penryn Campus, Penryn, Cornwall, TR10 9FE, United Kingdom; Medical Research Council-University of Glasgow Centre for Virus Research, School of Infection and Immunity, University of Glasgow, Glasgow, Scotland, G611QH, United Kingdom; Centre for Ecology and Conservation; Faculty of Environment, Science, and Economy; Biosciences, University of Exeter; Penryn Campus, Penryn, Cornwall, TR10 9FE, United Kingdom; Centre for Ecology and Conservation; Faculty of Environment, Science, and Economy; Biosciences, University of Exeter; Penryn Campus, Penryn, Cornwall, TR10 9FE, United Kingdom; Environment and Sustainability Institute; Centre for Ecology and Conservation, University of Exeter; Penryn Campus, Penryn, Cornwall, TR10 9FE, United Kingdom; Centre for Ecology and Conservation; Faculty of Environment, Science, and Economy; Biosciences, University of Exeter; Penryn Campus, Penryn, Cornwall, TR10 9FE, United Kingdom

**Keywords:** bacteriophage, phage therapy, comparative analyses, virulence, evolutionary biology, phylogeny, *Staphylococcaceae*, staphylococcus, virology, genotype-by-environment interactions

## Abstract

Predicting the outcomes of bacteria–phage interactions is a central challenge in microbial ecology, particularly across heterogeneous host environments. Evolutionary relatedness among bacteria can explain variation in phage susceptibility *in vitro*, but it remains unclear whether this predictive relationship persists in complex host environments and to what extent relatedness also explains variation in other bacterial traits, such as virulence. Here, we used an *in vivo* infection model to quantify how bacterial phylogeny and host environmental context mediate bacterial virulence and bacteria–phage interactions. We infected 4,608 *Galleria mellonella* larvae with 64 phylogenetically diverse *Staphylococcaceae* isolates, both with and without co-inoculation of the bacteriophage ISP, and recorded mortality and melanisation over 24 hours. We found that bacterial virulence varied among *Staphylococcaceae* strains and that a large proportion of this variation could be explained by the evolutionary relationships between bacteria, indicating that phylogeny may be useful in predicting bacterial virulence. The addition of phage significantly improved the survival of *G. mellonella* larvae, with an average 15.2% increase in endpoint survival and 10.1% reduction in endpoint melanisation across *Staphylococcaceae* strains. Unlike previous *in vitro* studies, the evolutionary relationships between bacterial isolates could not explain variation in *in vivo* phage efficacy across *Staphylococcaceae*. Concurrently, we found no evidence of a correlation between *in vivo* and *in vitro* measures of phage efficacy across bacterial isolates, highlighting the importance of the host environment in shaping bacteria–phage dynamics.

## Introduction

Predicting the outcomes of bacteria–phage interactions is a central challenge in microbial ecology. Bacteriophages (‘phage’) are abundant bacterial viruses that shape microbial communities by killing susceptible bacterial hosts, mediating competition, and driving reciprocal evolution of bacterial resistance and phage infectivity [1, 2]. These interactions are strongly context dependent, varying with bacterial genotype, community composition, and environmental conditions [3–5]. Evolutionary relatedness offers one potential source of predictability: closely related taxa often resemble one another in traits whose genetic determinants are predominantly inherited through common ancestry [6]. Across diverse systems, host and pathogen phylogenetic relationships can explain variation in infection traits such as virulence, transmissibility, and pathogen load [7–16], including bacterial susceptibility to phage *in vitro* [15, 17]. However, phylogenetic signals can depend on environmental context, as genotype-by-environment interactions can alter phenotypes among lineages and change or weaken patterns in trait expression across phylogenies [18]. It therefore remains unclear whether the predictive relationship between bacterial phylogeny and phage susceptibility persists in complex host environments, or whether evolutionary relatedness can also explain variation in other traits during bacterial infection, such as virulence.

Animal host environments differ from laboratory media in nutrient availability, pH, osmotic stress, spatial structure, microbial competition, and immune activity [19, 20], all of which can alter bacterial physiology [21–23]. These physiological changes can affect susceptibility to phage adsorption, infection, and replication [24–26], while phage virions can themselves be affected by the extracellular environment or targeted for clearance by the host immune system [27, 28]. During infection, bacteria and phage may also evolve in response to one another and to the host environment, allowing stochastic differences in evolutionary trajectories to contribute further to variation in infection outcomes [29, 30]. Together, these processes may alter the relative susceptibility of bacterial strains and disrupt phylogenetic patterns observed under simplified laboratory conditions. The applied importance of this problem is highlighted by phage therapy, where rising antimicrobial resistance has increased the need to identify phages that can control bacterial infections [31–33]. Phages are commonly selected using *in vitro* assays [34], but strong bacterial suppression *in vitro* does not consistently translate into reduced bacterial loads *in vivo* [34–37]. Similarly, experimental pre-adaptation of phages can improve bacterial suppression or host range *in vitro*, but evidence that these benefits reliably improve efficacy in animal hosts remains limited [38–41].

Variation in bacterial virulence provides an additional source of heterogeneity in animal–bacteria–phage infection outcomes. Virulence reflects the damage a pathogen causes to the animal host and can arise through multiple, non-mutually exclusive mechanisms, including bacterial growth, tissue invasion, toxin production, adhesion, and host immune-mediated pathology [42–44]. Bacterial strains with similar *in vitro* susceptibility to phage may nevertheless produce substantially different infection outcomes because they differ in the harm they cause to their host [45]. Phage-associated changes in infection outcome may reflect reduced bacterial burden, altered expression of virulence traits, altered host immune activation, or a combination of these processes [46, 47]. In some cases, lytic phage may even increase host damage through the release of endotoxins or other pathogen-associated molecular patterns (PAMPs) during bacterial lysis [48]. Explaining variation in virulence, both in the presence and absence of phage, is therefore important for predicting which bacterial infections are most likely to cause host damage and which are most likely to benefit from phage treatment.

Members of the *Staphylococcaceae* family occupy diverse host-associated niches and span commensal to opportunistic lifestyles [49–52], making them a tractable system for studying variation in virulence and phage susceptibility. Within this family, *Staphylococcus aureus* is a major cause of disease globally, producing infections ranging from relatively benign skin and soft-tissue infections to severe and life-threatening systemic disease [53, 54]. Bacterial phylogeny has previously been shown to explain a substantial proportion of the variation in phage susceptibility across *Staphylococcaceae in vitro*, suggesting that many determinants of susceptibility are vertically inherited in this system [48]. However, it is unknown whether this phylogenetic signal is preserved when *Staphylococcaceae-*ISP interactions occur within an animal host. It is likewise unclear whether bacterial phylogeny explains variation in *Staphylococcaceae* virulence during animal infection.

Here, we used a phylogenetically diverse panel of 64 *Staphylococcaceae* isolates, spanning 2 genera and 17 species, and the staphylococcal phage ISP [34, 55] to investigate bacterial virulence and phage efficacy *in vivo* using simultaneous bacteria–phage co-inoculation of *Galleria mellonella* larvae. *G. mellonella* provides a scalable *in vivo* infection model with a complex innate immune response, enabling comparative tests of host damage and phage effects across many bacterial strains [56, 57]. melanisation is a key component of this response, mediating pathogen encapsulation, reactive oxygen species production, and wound healing, with visible melanin accumulation providing a measure of immune activation and host damage over time [34, 55]. *G. mellonella* has previously been used to study staphylococcal infection phenotypes [58–60] and to evaluate antimicrobial [61, 62] and phage interventions [63–65]. Using this model, we address three questions: first, whether evolutionary relatedness explains variation in virulence across *Staphylococcaceae* during animal infection; second, whether evolutionary relatedness explains variation in ISP efficacy across bacterial strains during co-inoculation; and third, whether a standard *in vitro* measure of phage efficacy captures the same inter-strain patterns observed *in vivo.*

## Materials and methods

### *Staphylococcaceae* isolates

The *Staphylococcaceae* panel comprises 64 strains of *Staphylococcaceae*, representing a broad phylogenetic and geographic range of hosts. These strains span 2 genera and 17 species that were estimated to have last shared a common ancestor ~122 Ma [66]. Multi-locus sequencing typing (MLST) revealed that five Clonal Complexes (CC) of *S. aureus* were represented in this panel, including major complexes CC1 and CC8 (Table S1).

To minimise differences arising from laboratory adaptation, isogenic populations of each *Staphylococcaceae* strain had previously been isolated and stored in 25% glycerol at −80°C. When required, isolates were cultured by inoculating 10 ml of high-salt LB Miller broth (Formedium) in a sterile 30 ml glass universal with a scraping of the frozen culture and incubating at 37°C, 180 rpm overnight. To ensure that any observed differences in virulence or susceptibility to ISP were not a function of bacterial density, calibration curves using optical density (OD) and colony-forming units (CFUs)/ml were generated and used to dilute or concentrate each strain to a final concentration of 1 × 10^10^ CFU/ml prior to use.

### Inferring the host phylogeny

The methods used to infer the host phylogeny have been described in detail elsewhere [17]. Briefly, whole genome sequences were collected for 8 previously sequenced strains from NCBI (Table S1), and the remaining 56 sequences were obtained through whole genome sequencing. Library preparations and sequencing were performed by MicrobesNG (Birmingham, UK) and a core genome alignment of the isolates was constructed by identifying 102 orthologous genes shared between all 64 *Staphylococcaceae* isolates*.*

Ultrametric phylogenetic trees were constructed using BEAST v1.10 [67]. Sequence alignments were fitted to HKY substitution models using relaxed uncorrelated molecular clock models, gamma distributions of rate variation, and constant population size coalescent priors [67]. Separate substitution models and molecular clocks were fitted to 1^st^/2^nd^ and 3^rd^ codon positions to reflect differences in selective constraint [68]. Two independent Markov chain Monte Carlo (MCMC) chains were run until convergence was achieved, with a burn-in of <10%. Convergence of all parameters was checked using Tracer v1.6 [69].

### Bacteriophage

An isolate of ISP was kindly provided by Jean-Paul Pirnay and Maya Merabishvili at the Queen Astrid Military Hospital (Brussels, Belgium). ISP was propagated on the *S. aureus* strain EOE35 (hereafter referred to as the ‘propagation host’) and extracted using chloroform. The number of infectious phage present was quantified using plaque assays performed as previously described [17].

### Galleria mellonella

Final instar *G. mellonella* larvae were obtained from LiveFoods Direct (Sheffield, UK) and stored at 4°C in the dark to prevent pupation. Prior to injection, larvae were sorted into groups of 13 and stored in petri dishes at 4°C until required (maximum of 24 hours). Larvae with signs of injury, melanisation, or that were noticeably smaller or larger than average were excluded. The order in which individual larvae were assigned to each treatment was randomised to account for any subconscious biases in selection.

Body size has been shown to influence infection outcomes in *G. mellonella* [70]. To provide a measure of body size while minimising handling stress, a pilot set of 120 larvae were weighed and imaged (as described below) to establish the relationship between larval mass and pixel area. A linear model revealed a strong positive relationship between pixel area and weight (Pearson’s r = 0.94, *P* < .001; Fig. S1). Experimental larvae were then assigned weights based on the average of their pixel areas across images taken at each experimental timepoint, and weight was included as a covariate in all subsequent analyses.

### Inoculations

Bacteria were diluted to 1 × 10^10^ CFU/ml and phage to 1 × 10^10^ PFU/ml. For the without phage treatment, the appropriate bacterial strain was diluted 1:2 with LB Miller Broth to ensure an equivalent total injection volume and bacterial dose; 10 μL of the diluted bacteria was injected into the last left proleg of each larva using a 50 μL Hamilton syringe (Hamilton, Nevada, USA) for a final dose of 0.5 × 10^8^ bacteria. For the with phage treatment, bacteria were diluted 1:2 with phage immediately prior to injection, for a final dose of 0.5 × 10^8^ bacteria and 0.5 × 10^8^ phage (multiplicity of infection; MOI of 1). These doses were chosen based on pilot data that showed sufficient continuous variation in bacterial virulence and phage efficacy across strains at these doses within an experimental timeframe that minimised censoring due to larval pupation (Fig. S2).

Thirteen larvae were injected for each treatment to account for excessive post-injection bleeding, which occurred in a subset of individuals, and could alter the received dose of bacteria or phage. Larvae were left to recover for 15 minutes on filter paper before the 12 showing the least bleeding were transferred to each well of a 12-well plate and incubated at 37°C in the dark. Mortality—defined here as larvae being sessile and non-responsive to stimuli—was measured once an hour from 0- to 12-hours post infection (hpi), and at a final additional timepoint of 24 hpi. To minimise block effects and reduce experimental noise, bacterial treatments were randomized across the 10-day injection schedule. For each bacterial strain, 3 replicate groups of larvae were prepared, and on each day, 20 randomised combinations of bacterial strain and replicate were selected for injection. Both with and without phage treatments were performed on the same day for a given replicate. All inoculations were performed by a single individual to avoid introducing inter-operator variability.

Melanisation was quantified by photographing each plate at each hourly timepoint using a Panasonic Lumix GH3 camera fitted with a G Vario 14–45 mm f/3.5–5.6 lens. The camera was fixed with a tripod at 50 cm vertically from the plate surface, and RAW images were taken at a fixed focal length of 35 mm. Plates were evenly lit by overhead diffusion lighting and imaged on an 18% neutral grey background, which provided a consistent reference for white balance and exposure correction during image analysis. The bottom of each plate well was covered with a matte green cardboard disc created using a 2.2 cm diameter hole punch (Hobbycraft, UK) to allow for the separation of each larva from the background image by chroma keying. White balancing, segmentation, and quantification of melanisation were performed using a custom image processing Python script described in the Supplementary Methods S1 (see Fig. S3 for an example processed plate image).

To ensure any experimental observations were not due to pre-existing illness, injection trauma, or inoculum contamination, no-injection and injection controls (LB Miller broth or ISP only) were included on each day of injections. Control plates throughout the experiment showed no detectable mortality and little evidence of melanisation (Fig. S4).

### *In vitro* measures of phage efficacy

To compare phage efficacy measured *in vivo* and *in vitro*, optical density assays were used as a measure of bacterial growth suppression by phage under controlled *in vitro* conditions as previously described [17]. Briefly, each *Staphylococcaceae* isolate was incubated for 24 hours at 37°C in the presence or absence of bacteriophage ISP. Phage-treated cultures had final concentrations of 5 × 10^4^ CFU/ml bacteria and 2.5 × 10^3^ PFU/ml phage (MOI of 0.05), while control cultures contained bacteria only. The MOI used for the *in vitro* infections was lower than that used for the *in vivo* assays but was chosen based on a previous study that showed sufficient continuous non-saturating variation in phage efficacy across strains at these doses [17]. Three biological replicates were performed for each treatment condition. Following incubation, optical density was measured at 600 nm using a MultiSkan Sky Microplate Spectrophotometer (Thermo Fisher). *In vitro* phage efficacy was quantified as the proportional reduction in bacterial growth over 24 hours due to phage exposure, calculated as:


$$OD\ change=\frac{\left( OD\ with out\ phage- OD\ with\ phage\right)}{OD\ with out\ phage}$$

### Statistical analysis

To allow for the joint analysis of mortality and melanisation—which show different non-linear relationships with time—non-linear least squares models were used to provide summary parameter estimates that could be used as response variables in phylogenetic mixed models. These models were fitted separately to data from each plate of 12 larvae, yielding *n* = 3 independent replicates (plates) for each unique combination of bacterial isolate and ISP. Mortality data followed typical survival curve dynamics, but with curves frequently seen to reach a plateau below 100% mortality within the observational window (Fig. S5), indicating the presence of surviving individuals (i.e. a ‘cure fraction’). In some cases, mortality was low or absent during the 0–12 hour window but present at 24 hours, giving rise to two equally plausible survival curves: one in which the observed 24-hour mortality represents the upper asymptote of mortality (referred to here as the ‘cured’ assumption), and one in which it marks the onset of a larger, unobserved increase in mortality beyond the observational window (referred to here as the ‘unobserved’ assumption). All results and analyses presented below use parameter estimates inferred under the cured assumption, and parallel analyses performed using the unobserved assumption yielded qualitatively similar results. Melanisation tended to increase rapidly in the first few hours of infection, then slow to a plateau by the end of the observation window (Fig. S6). Michaelis–Menten curves were chosen to summarize these temporal dynamics, as they provide two parameters analogous to those of survival curves. In both cases, the fitted models provided estimates of an upper asymptote (maximum mortality or melanisation) and a T_50_ describing the time to half-maximal response. For both melanisation and mortality, the fitted models provided a close description of the observed data (Fig. S7).

Phylogenetic generalised linear mixed models were then used to investigate the extent to which the evolutionary relationships between bacteria could explain variation in the maximum and T_50_ parameter values for mortality and melanisation, and the change in these values due to the inclusion of ISP. A full description of the structure of these models, their run conditions, and equations used to calculate phylogenetic heritability and repeatability can be found in the Supplementary Methods S1. This two-stage approach allows phylogenetic patterns in mortality and melanisation dynamics to be analysed using specified non-linear curve forms, but does not propagate uncertainty in the within-plate estimates of maximum response and T_50_ into the downstream phylogenetic mixed models. This approach therefore assumes that uncertainty in these plate-level curve fits is small relative to the biological variation among strains, phage treatments, and biological replicates. All scripts and data used in this analysis are available at https://github.com/Sarah-K-Walsh/2026-In-Vivo-Phage-Efficacy.

## Results

### Bacterial virulence in *G. mellonella* is a phylogenetically heritable trait

To investigate whether the evolutionary relationships between bacteria could explain variation in their *in vivo* virulence and susceptibility to phage, we experimentally infected 4,608 *G. mellonella* larvae with 64 *Staphylococcaceae* isolates in the presence or absence of bacteriophage ISP and recorded mortality and melanisation over the following 24 hours (129,024 total observations).

**Figure 1:**
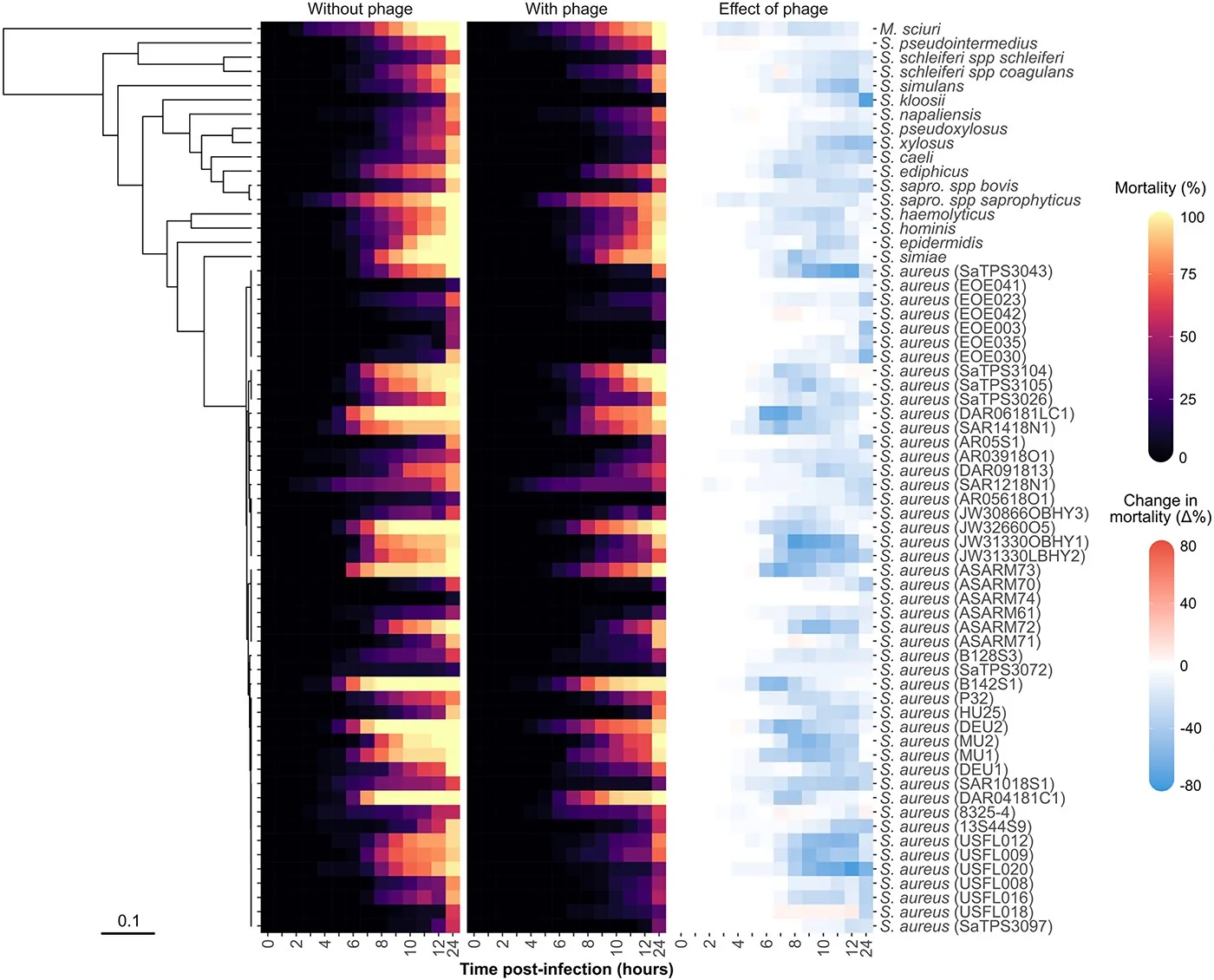
Mortality of *G. mellonella* larvae following *Staphylococcaceae* infection with and without co-inoculation of ISP. Heatmaps show percentage mortality over time (‘Without phage’ and ‘With phage’ panels) and change in percentage mortality with ISP (‘Effect of phage’ panel) for 64 *Staphylococcaceae* strains infecting *G. mellonella* larvae. Values shown are means across 3 biological replicates (plates of 12 individuals). A maximum clade credibility bacterial phylogeny is shown on the left, with a scale bar indicating substitutions-per-site.

**Figure 2:**
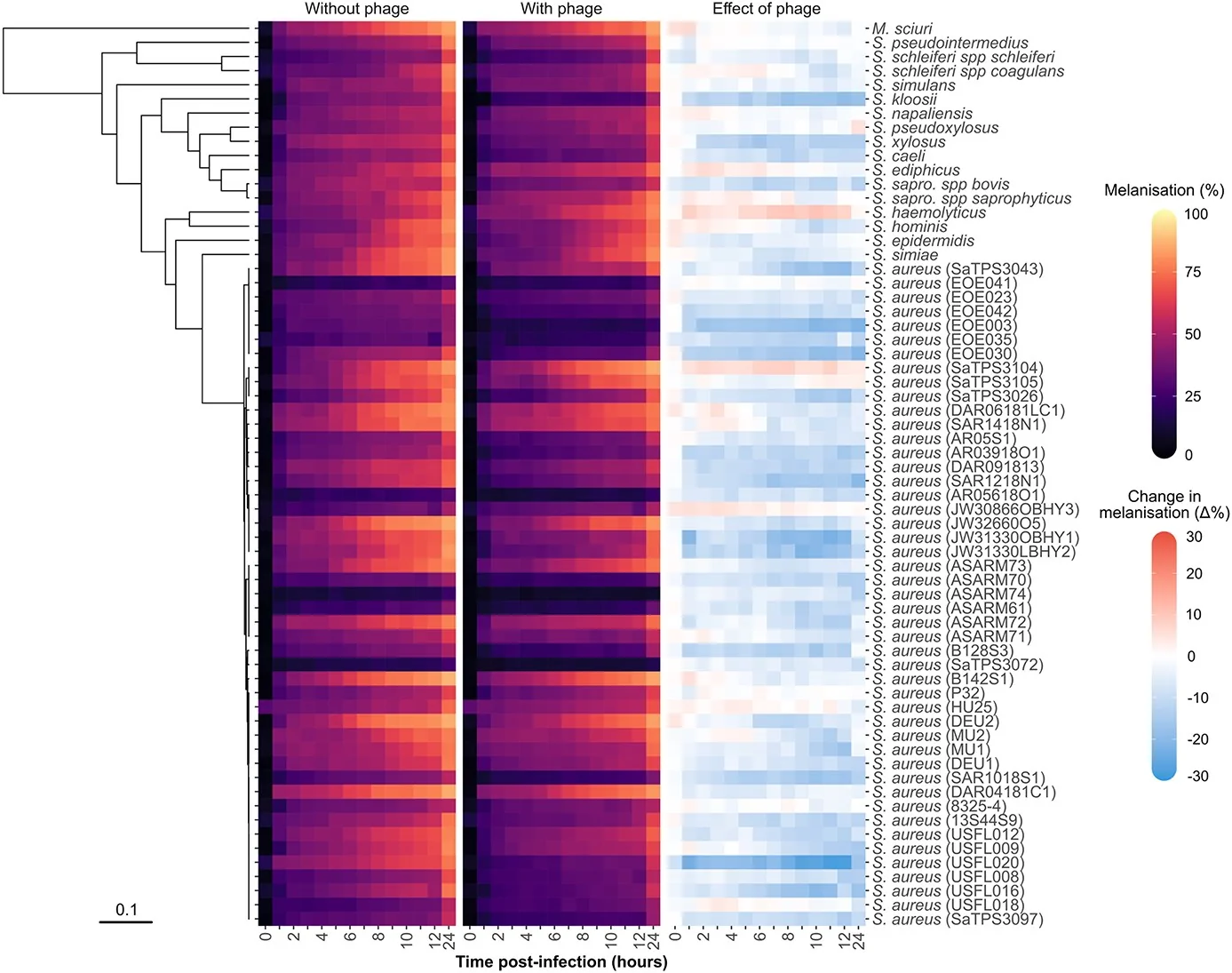
Melanisation in *G. mellonella* larvae following *Staphylococcaceae* infection with and without co-inoculation of ISP. Heatmaps show percentage melanisation over time (‘Without phage’ and ‘With phage’ panels) and change in percentage melanisation with ISP (‘Effect of phage’ panel) for 64 *Staphylococcaceae* strains infecting *G. mellonella* larvae. Values shown are means across 3 biological replicates (plates of 12 individuals). A maximum clade credibility bacterial phylogeny is shown on the left, with a scale bar indicating substitutions-per-site.

*G. mellonella* larvae showed considerable variation in their mortality and melanisation rates across the bacterial strains tested (Figs 1 and 2, respectively). Some bacterial strains caused mortality in all hosts within 8 hpi (e.g. *S. aureus* B142S1 and DEU2) while for other strains, a subset of hosts consistently survived infection beyond the observational window (e.g. *S. aureus* ASARM74 and EOE003). Similarly, some strains induced high levels of melanisation with either a rapid (e.g. *Mammaliicoccus sciuri*) or gradual onset (e.g. *S. schleiferi spp coagulans*) and others caused little melanisation (e.g. *S. aureus* EOE041 or *S. aureus* SaTPS3072).

**Figure 3:**
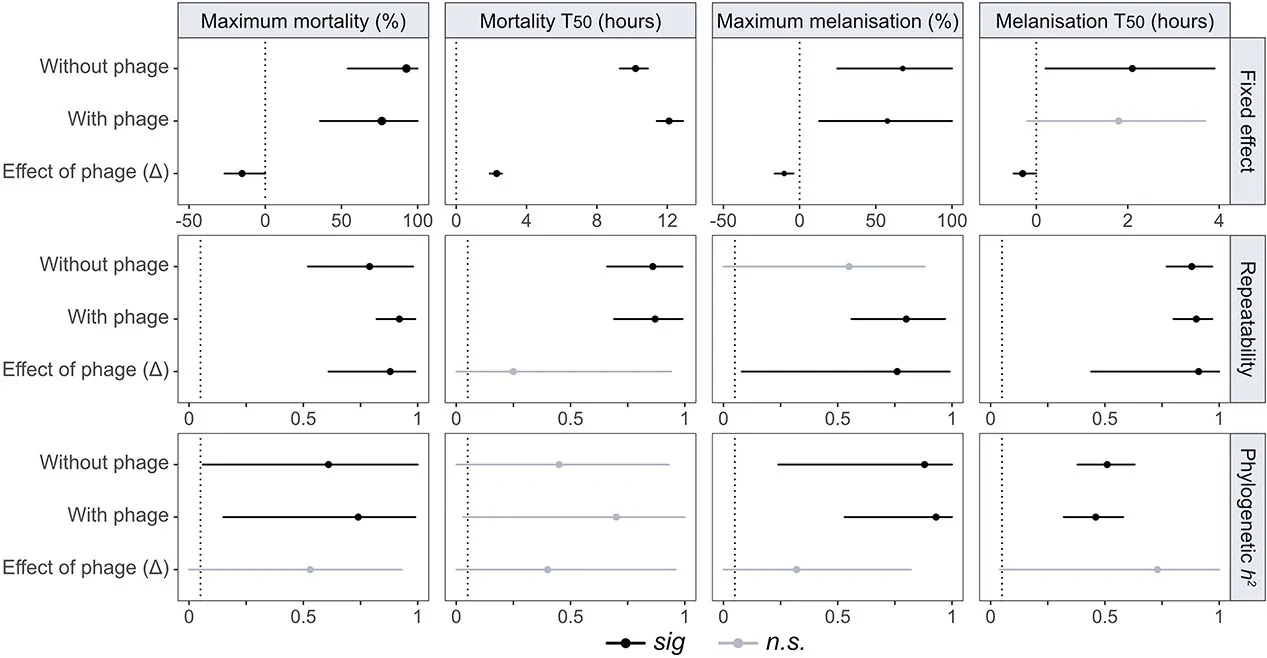
Estimates of fixed effects, repeatability, and phylogenetic heritability for mortality and melanisation parameters across *Staphylococcaceae* strains. Panels show the posterior means (points) and 95% CI intervals (lines) for phylogenetic mixed model estimates of inter-strain means (fixed effects), repeatability, and phylogenetic heritability of four virulence parameters: maximum mortality (%), mortality T_50_ (hours), maximum melanisation (%), and melanisation T_50_ (hours). Phylogenetic heritability estimates are taken from models of structure (1), while fixed effect and repeatability estimates are taken from models of structure (2). Dotted lines are used to highlight 0 (fixed effects) and 0.05 (repeatability, phylogenetic heritability). Significant (credible) estimates, whose 95% CI intervals do not contain these values, are highlighted in black.

Non-linear least squares models were used to summarise the dynamics of both mortality and melanisation over time, describing them in terms of their maximum (upper asymptote) and T_50_ (time to one-half of maximum). Across bacterial strains, mean maximum mortality was 92.6% (95% CIs: 54%–100%) with a mean T_50_ of 10.2 hours (95% CIs: 9.3–10.9 hours), suggesting that *Staphylococcaceae* infections are, in general, highly virulent in *G. mellonella*. Concurrently, the mean across-strain maximum melanisation was high (67.7%, 95% CIs: 25%–100%) with a mean T_50_ of 2.1 hours (95% CIs: 0.2–4 hours) indicating a rapid onset of the melanisation response. When these parameters were further analysed in phylogenetic mixed models, all four showed evidence of phylogenetic structure across *Staphylococcaceae*, with significant estimates of phylogenetic heritability detected for maximum mortality, maximum melanisation, and melanisation T_50_ (Fig. 3 ‘Without phage’ and Table S2). A significant estimate of repeatability, but not phylogenetic heritability, was detected for mortality T_50_ (Fig. 3 ‘Without phage’ and Table S2). Together, this suggests that a large proportion of the variation in virulence across *Staphylococcaceae* in *G. mellonella* larvae can be explained by the bacterial phylogeny.

### Phage efficacy varies *in vivo* but shows no detectable phylogenetic heritability

Co-inoculation of bacteria and ISP generally led to a decrease in both mortality (Fig. 1) and melanisation (Fig. 2), although considerable between-strain variation was again evident, with some strains showing large changes in infection dynamics with phage (e.g. *S. aureus* USFL020 and *S. kloosii*) and others appearing largely unaffected (e.g. *S. aureus* 8325-4 and *S. napaliensis*). When comparing the across-strain means with and without phage treatment, the 95% CI intervals of most mortality and melanisation parameters overlapped (Fig. 3 and Table S2). However, when analysed as a change in parameter value with the addition of phage (in effect treating the data as paired), significant across-strain mean effects of phage treatment were detected for all four parameters. These estimates suggest that, on average, the inclusion of ISP caused a 15.2% decrease in maximum mortality (95% CIs: 1%–27%), an increase of 3.2 hours to mortality T_50_ (95% CIs: 2.5–3.8 hours), a decrease in maximum melanisation of 10.1% (95% CIs: 4%–16.4%), and a decrease of 0.29 hours to melanisation T_50_ (95% CIs: 0.03–0.53 hours).

**Figure 4:**
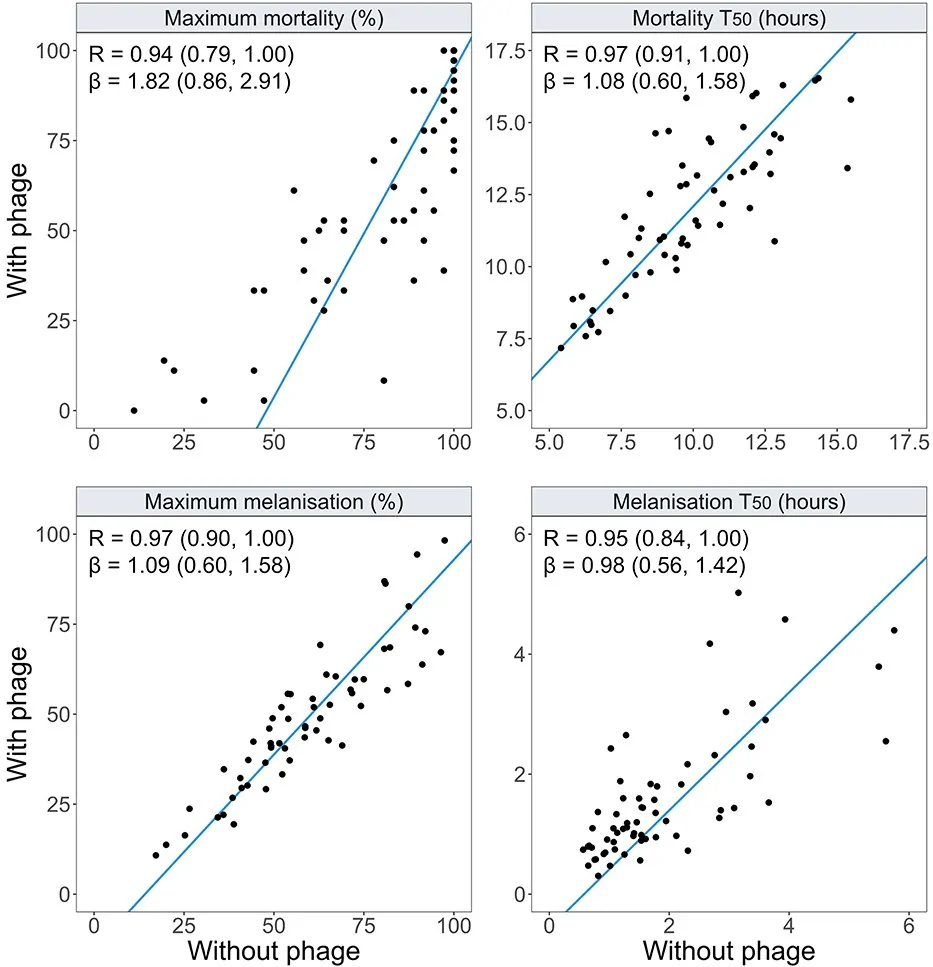
Inter-strain correlations between mortality and melanisation parameters in the presence and absence of ISP. Plots show the inter-strain correlations of the ‘With phage’ and ‘Without phage’ treatments for each mortality and melanisation parameter, with points indicating the mean value of each *Staphylococcaceae* strain. Trendlines, correlation coefficients, and slopes are taken from phylogenetic mixed models of structure (1), with trendlines whose coefficient and slope estimates do not include zero highlighted in blue.

The phylogenetic heritability in bacterial virulence remained consistent with the addition of phage (Fig. 3, ‘With phage’ and Table S2), with little change in the rank-order of virulence among bacterial strains in the presence or absence of ISP (Fig. 4). The addition of phage led to a small increase in phylogenetic variance for all tested parameters (Tables S3 and S4); however, this was not significant. In contrast, we found no detectable phylogenetic heritability for the effect of ISP on any mortality or melanisation parameters (Fig. 3, ‘Effect of phage’ and Table S2) with all estimates having broad credible intervals spanning much of the possible 0–1 range: maximum mortality (0.53, 95% CI: 0.00, 0.93), mortality T_50_ (0.40, 95% CI: 0.00, 0.96), maximum melanisation (0.32, 95% CI: 0.00, 0.82), and melanisation T_50_ (0.73, 95% CI: 0.04, 1.00). The phage-induced change in three of these parameters did show significant estimates of repeatability: maximum mortality (0.88, 95% CIs: 0.61–0.99), maximum melanisation (0.76, 95% CIs: 0.08–1.00), and melanisation T_50_ (0.91, 95% CIs: 0.44–1.00). Together, these results indicate that ISP efficacy is consistent within strains, but that any phylogenetic component of this variation was poorly estimated and not credibly distinguishable from zero in this dataset.

### *Staphylococcaceae* virulence traits are correlated across-strains

**Figure 5:**
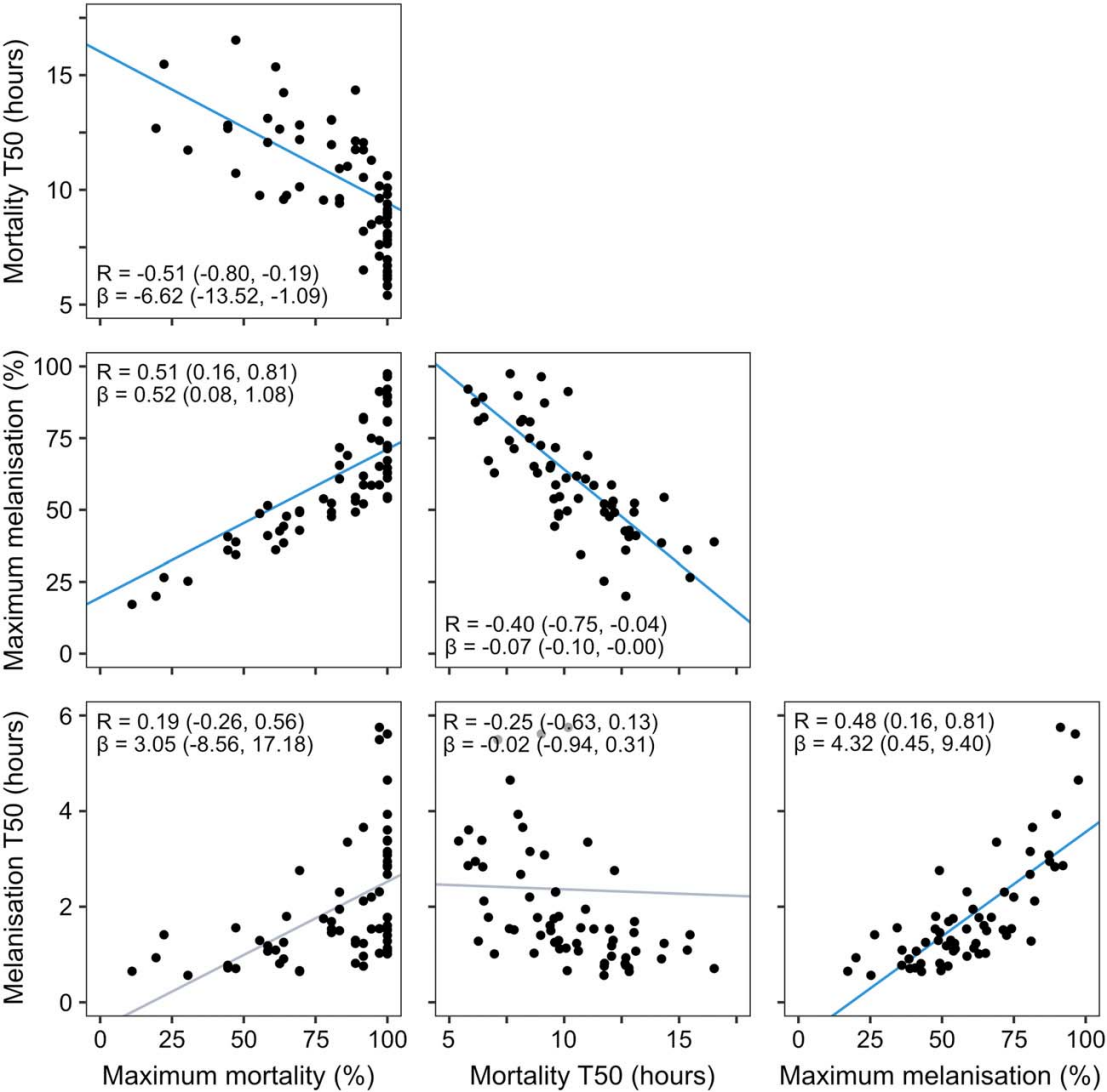
Inter-strain correlations between mortality and melanisation parameters in the absence of ISP. Plots show the inter-strain correlations of each pair of mortality and melanisation parameters, with points indicating the mean value of each *Staphylococcaceae* strain. Trendlines, correlation coefficients, and slopes are taken from phylogenetic mixed models of structure (1), with trendlines whose coefficient and slope estimates do not include zero highlighted in blue.

To investigate whether mortality and melanisation parameters were correlated across *Staphylococcaceae* strains, inter-strain correlations were estimated for each pair of parameters using phylogenetic mixed models (Fig. 5). Positive correlations were detected between maximum melanisation and melanisation T_50_ (R = 0.48, 95% CI: 0.16–0.81), and between maximum melanisation and maximum mortality (R = 0.51, 95% CI: 0.16–0.81), indicating that infections that induce greater endpoint melanisation tend to also induce longer melanisation responses and have higher endpoint mortality rates. Negative correlations were also detected between mortality T_50_ and maximum melanisation (R = −0.40, 95% CI: −0.04, −0.75), and between mortality T_50_ and maximum mortality (R = −0.51, 95% CI: −0.19, −0.80), suggesting that infections which cause rapid mortality are often also those with the highest endpoint mortality rates and melanisation responses. There were no detectable correlations between maximum mortality and melanisation T_50_ (R = 0.19, 95% CI: −0.26, 0.56), or between mortality T_50_ and melanisation T_50_ (R = −0.25, 95% CI: −0.63, 0.13), although the wide credible intervals indicate that weak-to-moderate associations cannot be excluded. The strength of all detected correlations remained consistent in the presence of ISP (Fig. S8).

### No detectable correlation in ISP efficacy *in vivo* and *in vitro*

Finally, we investigated whether ISP efficacy *in vivo*—measured as changes in mortality and melanisation parameters with phage treatment—showed any detectable correlation with efficacy *in vitro*, using optical density assays. We detected no detectable correlations between *in vivo* measures of ISP efficacy and *in vitro* OD-based efficacy (Fig. S9) with wide credible intervals spanning much of the possible correlation coefficient range. This indicates that any relationship between these measures was not strong enough to be detected in this dataset, consistent with the host environment altering the relative susceptibility of *Staphylococcaceae* strains to phage treatment.

## Discussion

Here, we examined whether *Staphylococcaceae* evolutionary relatedness explained variation in virulence and ISP phage susceptibility in an *in vivo G. mellonella* infection model. We found that bacterial virulence varied across strains and that the *Staphylococcaceae* phylogeny could explain a large proportion of this variation. Co-inoculation of ISP significantly reduced the maximum mortality, speed of mortality, and maximum melanisation experienced during infection, demonstrating that ISP was able to reduce bacterial virulence across a wide range of *Staphylococcaceae* isolates *in vivo*. However, there was no detectable phylogenetic heritability in *in vivo* phage efficacy and no correlation between phage efficacy *in vitro* and *in vivo*. This mismatch is consistent with genotype-by-environment interactions, whereby the host environment alters phage efficacy in a bacterial strain-specific way, weakening or obscuring the phylogenetically structured susceptibility observed *in vitro*.

Previously, we showed that the evolutionary relationships between staphylococcal hosts can explain variation in susceptibility to phage infection *in vitro* [17]. However, here we have shown that, *in vivo*, the relationship between bacterial relatedness and susceptibility to phage is less pronounced. This suggests that conditions within the *G. mellonella* host environment—such as immunity, local pH, or nutrient availability—interact with bacterial genotype to alter relative phage susceptibility across strains. These genotype-by-environment interactions may weaken or obscure the phylogenetic signal detected *in vitro*. Estimates of *in vivo* heritability and repeatability that were not detectably different from zero often had wide credible intervals, spanning much of the possible 0–1 range. This suggests that our dataset had limited power to differentiate weak from genuinely zero effects of phylogeny. However, because comparable models detected phylogenetic heritability for virulence in the present study and for *in vitro* phage susceptibility previously [17], any undetected phylogenetic component of *in vivo* phage efficacy is likely to be weaker than these effects.

Correspondingly, no inter-strain correlations were detected between any *in vivo* and *in vitro* metrics of phage efficacy, indicating that the host environment can act as a strong source of variation that disrupts *in vitro* phylogenetic heritability. In applied settings, phage are typically selected using *in vitro* screens of bacterial growth suppression (often complemented by plaque-based or molecular readouts), an approach that implicitly assumes laboratory efficacy will translate to performance *in vivo*. Notably, in previous work, we observed strong positive correlations between OD assays and both qPCR and plaque assays (recorded as a binary trait) [17], suggesting that these approaches capture broadly consistent aspects of phage efficacy *in vitro*. However, because we did not directly compare these alternative *in vitro* assays with the *in vivo* outcomes measured here, we cannot exclude the possibility that other assays would show different relationships with *in vivo* susceptibility. Nevertheless, over-reliance on individual *in vitro* assays to select phage for therapy may explain some of the observed inconsistency in *in vivo* phage efficacy, with phage showing highly variable success rates in treating similar infections [34].

Phylogenetic approaches have shown that evolutionary relatedness among both hosts and pathogens can explain variation in infection traits [7–16]. However, although bacterial virulence has been associated with particular phylogenetic clades [71–73], including within *S. aureus* [74–76]*,* formal tests quantifying how much variation in virulence is explained by bacterial phylogeny remain rare. Here, we find that the evolutionary relationships between bacteria can explain a considerable proportion of their virulence *in vivo*, with closely related strains of bacteria tending to show similar levels of virulence. This similarity in virulence among close relatives is likely underpinned by shared genetic determinants of infection, including variation in within-host persistence and replication, virulence factor repertoires, and regulation of virulence expression (e.g. quorum sensing) [77–79]. Conversely, we do observe some instances where closely related species show large differences in their virulence (e.g. the *S. aureus* strains EOE041, EOE023, and EOE003), suggesting that there are instances where Brownian evolutionary processes alone cannot explain variation in virulence. This may reflect evolutionarily recent changes in the genetic components that influence virulence (e.g. single-nucleotide polymorphisms in virulence factors [80, 81]) or the loss or gain of genetic components in a non-vertically inherited way (e.g. horizontal gene transfer leading to the acquisition of a novel virulence factor [82, 83]). Improving our understanding of how the relatedness of bacterial strains can explain their virulence, and the taxonomic scales at which this relationship breaks down, may improve our ability to predict bacterial virulence, disease outcome, and manage treatment plans in a clinical setting.

The mechanisms by which ISP reduces mortality caused by some bacterial strains during co-inoculation remain uncertain. Notably, as ISP and bacteria were administered simultaneously, the effects described here should be interpreted as phage-associated changes during infection establishment rather than treatment of an established infection. These effects could plausibly reflect bacterial killing, delayed bacterial growth, altered expression of virulence traits, altered host immune activation, differences in phage persistence or clearance, or the emergence of phage-resistant bacteria during infection. Similarly, phage-associated changes in melanisation may reflect changes in bacterial burden, virulence expression, immune activation, or exposure to bacterial products released during lysis. Previous studies have shown that phage-mediated bacterial lysis can increase host damage by releasing immunostimulatory PAMPs [48], and the increased melanisation observed for a minority of strains following ISP co-inoculation may suggest that similar processes contribute to infection outcome in this system. Future work should seek to resolve these mechanisms, particularly given the continued development of ISP as a standardized therapeutic phage [34].

Host immune responses can influence bacterial populations and, by extension, phage dynamics, through various mechanisms including direct effects on bacterial or phage survival, and indirect effects on bacterial susceptibility to phage infection [84, 85]. The innate immune system has been shown to play a role in the clearance of bacteriophage *in vivo* [86, 87], including neutralisation by the complement system [88, 89] or phagocytosis [87, 90]. In our data, we see several instances in which the addition of phage led to a marked early increase in *G. mellonella* survival compared to the no-phage treatment, with no corresponding improvement in endpoint survival (e.g. *S. aureus* DAR06181LC1), a pattern that may be consistent with phage clearance within 24 hours of inoculation. Repeated or sustained dosing could modify these dynamics by maintaining phage densities above thresholds required for productive infection and *in situ* amplification, consistent with pharmacokinetic/pharmacodynamic work on density-dependent phage amplification and clearance [91, 92]. However, it has also been observed that repeated dosing of phage can lead to lower serum phage titres and increased host immune clearance [93, 94]. In this system, repeated dosing was not explored because it would require multiple injections per larva, which introduces injection-related mortality and confounds survival outcomes in *G. mellonella.*

Together, our results demonstrate that the *Staphylococcaceae* phylogeny is a strong predictor of bacterial virulence *in vivo*. Furthermore, we highlight that *in vivo* ISP efficacy shows no detectable correlation with an *in vitro* measure of ISP efficacy, suggesting alternative approaches may be required to predict phage treatment outcome. While *G. mellonella* provides a scalable and experimentally tractable animal infection model, it is not a direct surrogate for mammalian host environment. Although larvae can be incubated at 37°C, temperature can influence both bacterial physiology and insect immune responses, and the *Galleria* immune system differs substantially from mammalian immunity, including the absence of adaptive antibody-mediated immune responses and differences in phage clearance mechanisms. The patterns observed here should therefore be interpreted as evidence that host-associated environments can alter bacteria–phage outcomes, but their direct relevance to mammalian therapy will require validation in mammalian infection models. More broadly, it remains unclear whether bacterial phylogeny and *in vitro* measures of phage susceptibility can predict bacteria–phage interactions in other complex natural environments, including soils, aquatic systems, and polymicrobial communities. Resolving this question will be important for understanding how phages contribute to changes in microbial community composition and function across environments. Future work should therefore test the predictive value of bacterial phylogeny and *in vitro* assays across a wider range of environmental contexts, while also resolving the mechanisms by which ISP alters bacterial virulence *in vivo*.

## Supplementary Material

V011_Galleria_SupplementaryMaterials_Clean_ycag232

## Data Availability

All of the datasets and scripts used for these analyses are publicly available on GitHub (https://github.com/Sarah-K-Walsh/2026-In-Vivo-Phage-Efficacy).
