# Supplementary material for: Detectable effects of bacterial phylogeny on virulence but not phage efficacy in an in vivo Galleria mellonella model
Source: ISME Commun. 2026 Aug 20;6(1):ycag232. doi: 10.1093/ismeco/ycag232 (PMC13615537; doi:10.1093/ismeco/ycag232)
Supplement: Supplementary file 1 [file v011_galleria_supplementarymaterials_clean_ycag232.docx]

**Supplementary Materials**

**Detectable effects of bacterial phylogeny on virulence but not phage efficacy in an *in vivo Galleria mellonella* model**

**Running title: *In vivo* patterns in bacteria-ISP traits**

Sarah K. Walsh^1,2*^, Ryan M. Imrie^1,2^, Angus Buckling^2,3^, and Ben Longdon^2^

^1^_­_Medical Research Council-University of Glasgow Centre for Virus Research; University of Glasgow, Glasgow, Scotland; G61 1QH; UK.

^2^Centre for Ecology and Conservation; Faculty of Environment, Science, and Economy; Biosciences; University of Exeter; Penryn Campus; Penryn; Cornwall; TR10 9FE; UK.

^3^Environment and Sustainability Institute; University of Exeter; Penryn Campus; Penryn; TR10 9FE; Cornwall; UK.

* Corresponding author: [sarah.walsh.2@glasgow.ac.uk](mailto:sarah.walsh.2@glasgow.ac.uk), Dr. Sarah K Walsh, MRC-University of Glasgow Centre for Virus Research, Sir Michael Stocker Building, 464 Bearsden Road, Glasgow, Scotland, G61 1QH.

**Supplementary Methods**

Image processing and calculation of melanisation

Following image acquisition, RAW (.RW2) images were processed using a custom Python script. RAW files were first demosaiced and converted to RGB, then white balance and exposure were normalised using the neutral grey card included in each image. Plate wells were identified by an automated circle-finding algorithm: green-dominant pixels were first identified by comparing RGB channel intensities, generating a binary mask corresponding to the green well inserts. This mask was refined using iterative morphological erosion and dilation to remove noise and fill gaps. For each well, a fixed-radius circular mask was placed on an approximate starting centre coordinate, and the location of this mask was varied iteratively in all cardinal and diagonal directions – first in large (20px), then progressively smaller steps (10px, 5px, 2px, 1px) – to maximise overlap between the circular mask and the green-dominant pixels of the well. Circular masks were then applied to extract individual cropped images of each well.

Within each cropped well image, larvae were segmented from the green well inserts by identifying red-dominant (i.e., low-green) pixels. The largest single connected component in each well was assumed to be the larva and retained, and the boundaries of the larva mask were refined using morphological filtering before being applied. False colour validation images (Figure S2) were generated for each plate showing the positions of well and larva masks. These images were manually inspected to detect any poor segmentation with the automated script. In a small number of cases, haemolymph leakage onto the green well insert caused larva masks to include a large proportion of dark-green background. In these cases, the larva masks were manually adjusted in image editing software (Affinity Photo 2) to remove the well background. For each segmented larva, the total number of pixels was recorded as a proxy for larval size, and mean RGB values were extracted and converted to a greyscale luminance value, with melanisation then calculated as $1-luminance$. The minimum and maximum observed melanisation values were then used as bounds to define 0-100% melanisation within the context of this experiment.

Phylogenetic Generalised Linear Mixed Model Structures

Phylogenetic generalised linear mixed models were used to investigate the extent to which the evolutionary relationships between bacteria could explain variation in the maximum and T_50_ parameter values for mortality and melanisation, and the change in these values due to the inclusion of ISP.

The structures of these models were as follows:

$y_{\Phi bit}=\beta_{1:\Phi t}+\mu_{p:\Phi t}+\mu_{b:\Phi t}+\mu_{r:\Phi}+e_{\Phi bit}$ (1)

$y_{\Phi bit}=\beta_{1:\Phi t}+\mu_{p:\Phi t}+\mu_{r:\Phi}+e_{\Phi bit}$ (2)

In these models, $y_{\Phi bit}$ is the parameter estimate of trait *t* (maximum mortality or melanisation; T_50_ mortality or melanisation; or larval weight) in infection condition *Φ* (with phage; without phage; effect of phage Δ) for the *ith* biological replicate of bacterial strain *b*. The fixed effect $\beta_{1}$ is the intercept for each trait; the random effect $\mu_{p}$ represents the effect of host phylogeny under a Brownian model of evolution; $\mu_{b}$ represents a categorical bacterial-strain-specific effect that is independent of phylogeny and explicitly estimates the non-phylogenetic component of trait variance. Lastly, $\mu_{r}$ represents a random effect of experimental block, and $e$ is the model residuals. All random effects and residuals were assumed to be Gaussian distributed with a centred mean of 0.

The proportion of between-strain variance that can be explained by the bacterial phylogeny was calculated from model (1) using the equation $\frac{V_{p}}{V_{p}+V_{b}}$, which is equivalent to phylogenetic heritability or Pagel’s lambda [1, 2]. Here, $V_{p}$ and $V_{b}$ represent the phylogenetic and bacteria strain-specific components of trait variance respectively [3]. The repeatability of traits within-bacterial-strains after accounting for any phylogenetic variance was calculated as $\frac{V_{p}}{V_{p}+V_{e}}$, where $V_{p}$ and $V_{e}$ are the phylogenetic and residual variance components from model (2). Inter-strain correlation coefficients were calculated from model $V_{p}$ matrices ${cov}_{x,y}\sqrt{{var}_{x}\times{var}_{y}}$, and correlation slopes as $\frac{{cov}_{x,y}}{{var}_{x}}$. For parameters on an unbounded scale (correlation coefficients, slopes), effects were considered significant when the 95% credible intervals did not include zero. For proportions of variance (phylogenetic heritability, repeatability), effects were considered non-zero when the lower bound of the 95% credible interval exceeded a value of 0.05. This threshold was chosen to avoid treating effects whose posterior distributions allocate a high probability mass near zero as meaningful. For simplicity, credible effects are referred to as ‘significant’ throughout the remainder of this text.

Models were fitted using the R package MCMCglmm [4] in R v4.5.2 [5], and were run for 13 million MCMC generations, sampled every 5,000 iterations with a burn-in of 3 million generations. Parameter expanded priors were placed on the covariance matrices, resulting in multivariate F distributions with marginal variance distributions scaled by 1,000. Inverse-gamma priors were placed on the residual variances, with a shape and scale equal to 0.02. To ensure the model outputs were robust to changes in prior distribution, models were also fitted with flat and inverse-Wishart priors, which gave qualitatively similar results. Parameter estimates reported are means of the posterior density, and 95% credible intervals (CIs) were taken to be the 95% Highest Posterior Density (HPD) intervals. All scripts and data used in this analysis are available at <https://github.com/Sarah-K-Walsh/2026-In-Vivo-Phage-Efficacy>.

**Supplementary Tables**

**Table S1:** ***Staphylococcaceae* samples metadata.** Clonal complex (CC) and sequence type (ST), where available, were determined using PubMLST. *S. aureus* strains missing an ST are coagulase-negative and therefore do not have an MLST scheme and the *S. aureus* strains with STs but no CCs are not similar enough to other strains to be assigned a CC. In ‘source’, ‘JPP’ is John-Paul Pirnay, ‘EF’ is Ed Feil, and ‘GP’ is Gavin Paterson.

| **Isolate ID** | **Species** | **Host** | **Country** | **Year** | **CC** | **ST** | **NCBI BioSample ID** | **Source** |
| --- | --- | --- | --- | --- | --- | --- | --- | --- |
| 13S44S9 | *S. aureus* | *Homo sapiens* | BEL | 2012 | 8 | 8 | SAMN31484028 | JPP |
| 271Y | *S. kloosii* | *Eptesicus serotinus* | GBR | 2015 | --- | --- | SAMN31484029 | EF |
| 2111F7LW | *M. sciuri* | *Pteropus livingstonii* | GBR | 2017 | --- | --- | SAMN31484030 | EF |
| 27420LC | *S. simiae* | *Pteropus livingstonii* | GBR | 2016 | --- | --- | SAMN31484031 | EF |
| 2745SW | *S. nepalensis* | *Pteropus livingstonii* | GBR | 2016 | --- | --- | SAMN31484032 | EF |
| 82B | *S. caeli* | Environmental | ITA | 2013 | --- | --- | SAMEA2297795 | GP |
| 8325-4 | *S. aureus* | *---* | --- | --- | 8 | 8 | SAMN31484033 | EF |
| AR03918O1 | *S. aureus* | *Sciurus carolinensis* | GBR | 2018 | --- | 133 | SAMN31484034 | EF |
| AR05S1 | *S. aureus* | *Sciurus carolinensis* | GBR | 2015 | --- | --- | SAMN31484035 | EF |
| AR05618O1 | *S. aureus* | *Sciurus carolinensis* | GBR | 2018 | --- | 49 | SAMN31484036 | EF |
| ASARM61 | *S. aureus* | *Homo sapiens* | GBR | 2006 | 22 | 22 | SAMN31484037 | EF |
| ASARM70 | *S. aureus* | *Homo sapiens* | GBR | 2006 | 22 | 22 | SAMN31484038 | EF |
| ASARM71 | *S. aureus* | *Homo sapiens* | GBR | 2006 | 22 | 22 | SAMN31484039 | EF |
| ASARM72 | *S. aureus* | *Homo sapiens* | GBR | 2006 | 22 | 22 | SAMN31484040 | EF |
| ASARM73 | *S. aureus* | *Homo sapiens* | GBR | 2006 | 22 | 22 | SAMN31484041 | EF |
| ASARM74 | *S. aureus* | *Homo sapiens* | GBR | 2006 | 22 | 22 | SAMN31484042 | EF |
| NCTC7692 | *S. saprophyticus subsp. saprophyticus* | Environmental | --- | 1948 | --- | --- | SAMEA3517999 | GP |
| NCTC11320 | *S. hominis spp hominis* | *Homo sapiens* | USA | 1975 | --- | --- | SAMEA3539708 | GP |
| NCTC11043 | *S. xylosus* | *Homo sapiens* | USA | 1975 | --- | --- | SAMEA3539705 | GP |
| B128S3 | *S. aureus* | *Sciurus carolinensis* | GBR | 2015 | 1 | 188 | SAMN31484043 | EF |
| B142S1 | *S. aureus* | *Sciurus carolinensis* | GBR | 2015 | --- | 692 | SAMN31484044 | EF |
| DAR04181C1 | *S. aureus* | *Cervus elaphus* | GBR | 2018 | 8 | 1958 | SAMN31484045 | EF |
| DAR06181LC1 | *S. aureus* | *Cervus elaphus* | GBR | 2018 | --- | 3237 | SAMN31484046 | EF |
| DAR091813 | *S. aureus* | *Cervus elaphus* | GBR | 2018 | --- | 425 | SAMN31484047 | EF |
| DEU1 | *S. aureus* | *Homo sapiens* | TUR | 2009 | 8 | 239 | SAMN31484048 | EF |
| DEU2 | *S. aureus* | *Homo sapiens* | TUR | 2009 | 8 | 239 | SAMN31484049 | EF |
| DSM104441 | *S. edaphicus* | Environmental | ATA | 2013 | --- | --- | SAMN31484050 | GP |
| DSM107950 | *S. pseudoxylosus* | *Bos taurus* | FRA | 2002 | --- | --- | SAMN31484051 | GP |
| DSM18669 | *S. saprophyticus subsp. Bovis* | *Bos taurus* | CZE | 1996 | --- | --- | SAMN31484052 | GP |
| DSM21284 | *S. pseudo-intermedius* | *Felis catus* | BEL | 2008 | --- | --- | SAMN31484053 | GP |
| NCTC12218 | *S. schleiferi subsp. coagulans* | *Homo sapiens* | --- | 1988 | --- | --- | SAMEA3221103 | GP |
| DSM6628 | *S. schleiferi subsp. schleiferi* | *Canis lupus* | --- | 1991 | --- | --- | SAMN31484054 | GP |
| EOE23 | *S. aureus* | *Homo sapiens* | GBR | 1998 | 30 | 36 | SAMN31484055 | EF |
| EOE03 | *S. aureus* | *Homo sapiens* | GBR | 1998 | 30 | 3488 | SAMN31484056 | EF |
| EOE30 | *S. aureus* | *Homo sapiens* | GBR | 1998 | 30 | 36 | SAMN31484057 | EF |
| EOE35 | *S. aureus* | *Homo sapiens* | GBR | 2003 | 30 | 36 | SAMN31484058 | EF |
| EOE41 | *S. aureus* | *Homo sapiens* | GBR | 2005 | 30 | 36 | SAMN31484059 | EF |
| EOE42 | *S. aureus* | *Homo sapiens* | GBR | 2005 | 30 | 36 | SAMN31484060 | EF |
| HU25 | *S. aureus* | *Homo sapiens* | BRA | 1905 | 8 | 239 | SAMN31484061 | EF |
| JW32660O5 | *S. aureus* | *Sciurus carolinensis* | GBR | 2018 | --- | 49 | SAMN31484062 | EF |
| JW30866O BHY3 | *S. aureus* | *Sciurus carolinensis* | GBR | 2018 | --- | 49 | SAMN31484063 | EF |
| JW31330L BHY2 | *S. aureus* | *Sciurus carolinensis* | GBR | 2018 | --- | 49 | SAMN31484064 | EF |
| JW31330O BHY1 | *S. aureus* | *Sciurus carolinensis* | GBR | 2018 | --- | 49 | SAMN31484065 | EF |
| MU1 | *S. aureus* | *Homo sapiens* | TUR | 2010 | 8 | --- | SAMN31484066 | EF |
| MU2 | *S. aureus* | *Homo sapiens* | TUR | 2010 | 8 | 239 | SAMN31484067 | EF |
| NCTC11042 | *S. haemolyticus* | *Homo sapiens* | CZE | 1976 | --- | --- | SAMEA3233544 | GP |
| NCTC11046 | *S. simulans* | *Homo sapiens* | CZE | 1976 | --- | --- | SAMEA3504572 | GP |
| NCTC11047 | *S. epidermidis* | *Homo sapiens* | CZE | 1976 | --- | 5 | SAMEA3233545 | GP |
| P32 | *S. aureus* | *Homo sapiens* | POL | 1996 | 8 | 239 | SAMN31484068 | EF |
| SaTPS3026 | *S. aureus* | *Homo sapiens* | AUS | 2013 | 93 | 93 | SAMN31484069 | EF |
| SaTPS3043 | *S. aureus* | *Homo sapiens* | AUS | 2013 | 30 | 30 | SAMN31484070 | EF |
| SaTPS3072 | *S. aureus* | *Homo sapiens* | AUS | 2013 | 1 | 1 | SAMN31484071 | EF |
| SaTPS3097 | *S. aureus* | *Homo sapiens* | AUS | 2013 | 8 | 8 | SAMN31484072 | EF |
| SaTPS3104 | *S. aureus* | *Homo sapiens* | AUS | 2013 | 93 | 93 | SAMN31484073 | EF |
| SaTPS3105 | *S. aureus* | *Homo sapiens* | AUS | 2013 | 93 | 93 | SAMN31484074 | EF |
| SAR1018S1 | *S. aureus* | *Ovis aries* | GBR | 2018 | 8 | 8 | SAMN31484075 | EF |
| SAR1218N1 | *S. aureus* | *Ovis aries* | GBR | 2018 | --- | 1640 | SAMN31484076 | EF |
| SAR1418N1 | *S. aureus* | *Ovis aries* | GBR | 2018 | --- | 130 | SAMN31484077 | EF |
| USFL008 | *S. aureus* | *Homo sapiens* | USA | 2009 | 8 | 8 | SAMN31484078 | EF |
| USFL009 | *S. aureus* | *Homo sapiens* | USA | 2009 | 8 | 8 | SAMN31484079 | EF |
| USFL012 | *S. aureus* | *Homo sapiens* | USA | 2009 | 8 | 8 | SAMN31484080 | EF |
| USFL016 | *S. aureus* | *Homo sapiens* | USA | 2009 | 8 | 8 | SAMN31484081 | EF |
| USFL018 | *S. aureus* | *Homo sapiens* | USA | 2009 | 8 | 8 | SAMN31484082 | EF |
| USFL020 | *S. aureus* | *Homo sapiens* | USA | 2009 | 8 | 8 | SAMN31484083 | EF |

**Table S2:** **Estimates of fixed effects, repeatability, and phylogenetic heritability for mortality and melanisation parameters across *Staphylococcaceae* strains.** The table shows the posterior means (points) and 95% highest-posterior density (HPD) intervals for phylogenetic mixed model estimates of inter-strain means (fixed effects), repeatability, and phylogenetic heritability of four virulence parameters: maximum mortality (%), mortality T_50_ (hours), maximum melanisation (%), and melanisation T_50_ (hours). Phylogenetic heritability estimates are taken from models of structure (1), while fixed effect and repeatability estimates are taken from models of structure (2). Credible estimates, whose HPD intervals do not contain 0 (fixed effects) and 0.05 (repeatability, phylogenetic heritability) values, are highlighted in black.

| **Condition** | **Parameter** | **Fixed effect** | **Phylogenetic *h^2^*** | **Repeatability** |
| --- | --- | --- | --- | --- |
| Without phage | Maximum mortality (%) | **92.6 (54.1, 100)** | **0.61 (0.06, 1.00)** | **0.79 (0.52, 0.98)** |
|  | Mortality T50 (hours) | **10.2 (9.3, 10.9)** | 0.45 (0.00, 0.93) | **0.86 (0.66, 0.99)** |
|  | Maximum melanisation (%) | **67.7 (24.7, 100)** | **0.88 (0.24, 1.00)** | 0.55 (0.00, 0.88) |
|  | Melanisation T50 (hours) | **2.1 (0.2, 3.9)** | **0.51 (0.38, 0.63)** | **0.88 (0.77, 0.97)** |
| With phage | Maximum mortality (%) | **76.5 (35.9, 100)** | **0.74 (0.15, 0.99)** | **0.92 (0.82, 0.99)** |
|  | Mortality T50 (hours) | **12.1 (11.4, 12.9)** | 0.70 (0.03, 1.00) | **0.87 (0.69, 0.99)** |
|  | Maximum melanisation (%) | **57.6 (12.8, 100)** | **0.93 (0.53, 1.00)** | **0.80 (0.56, 0.97)** |
|  | Melanisation T50 (hours) | 1.8 (-0.2, 3.6) | **0.46 (0.32, 0.58)** | **0.90 (0.80, 0.97)** |
| Effect of phage (delta) | Maximum mortality (%) | **-15.2 (-26.8, -0.1)** | 0.53 (0.00, 0.93) | **0.88 (0.61, 0.99)** |
|  | Mortality T50 (hours) | **2.3 (1.9, 2.6)** | 0.40 (0.00, 0.96) | 0.25 (0.00, 0.94) |
|  | Maximum melanisation (%) | **-10.1 (-16.4, -4.0)** | 0.32 (0.00, 0.82) | **0.76 (0.08, 0.99)** |
|  | Melanisation T50 (hours) | **-0.3 (-0.5, -0.0)** | 0.73 (0.04, 1.00) | **0.91 (0.44, 1.00)** |

**Table S3:** **Phylogenetic, strain-specific, and residual variances for mortality and melanisation parameters across *Staphylococcaceae* strains.** The table shows the posterior means (points) and 95% highest-posterior density (HPD) intervals for the phylogenetic variance (*V_p_*), strain-specific variance (*V_b_*), and residual variance (*e*) taken from Model 1, used to calculate phylogenetic heritability.

| **Condition** | **Parameter** | **Phylogenetic variance (*V_p_*)** | **Strain-specific variance (*V_b_*)** | **Residual variance (*e*)** |
| --- | --- | --- | --- | --- |
| Without phage | Maximum mortality (%) | 4.25 (0.00, 14.05) | 1.49 (0.00, 3.16) | 1.32 (0.51, 2.06) |
|  | Mortality T50 (hours) | 5.02 (0.00, 21.80) | 1.94 (0.35, 3.73) | 0.98 (0.01, 2.27) |
|  | Maximum melanisation (%) | 7.99 (0.00, 28.26) | 0.16 (0.00, 0.45) | 1.16 (0.61, 1.71) |
|  | Melanisation T50 (hours) | 11.52 (0.89, 26.57) | 10.97 (1.68, 24.62) | 1.38 (0.96, 1.82) |
| With phage | Maximum mortality (%) | 18.42 (0.00, 51.79) | 2.98 (0.46, 5.91) | 1.78 (0.96, 2.67) |
|  | Mortality T50 (hours) | 8.73 (0.00, 33.47) | 0.95 (0.00, 2.75) | 4.45 (2.79, 6.27) |
|  | Maximum melanisation (%) | 11.05 (0.00, 35.24) | 0.11 (0.00, 0.36) | 0.72 (0.28, 1.17) |
|  | Melanisation T50 (hours) | 10.97 (1.68, 24.62) | 12.70 (1.70, 26.07) | 1.23 (0.85, 1.65) |

**Table S4:** **Phylogenetic and residual variances for mortality and melanisation parameters across *Staphylococcaceae* strains.** The table shows the posterior means (points) and 95% highest-posterior density (HPD) intervals for the phylogenetic variance (*V_p_*) and residual variance (*e*) taken from Model 2, used to calculate repeatability.

| **Condition** | **Parameter** | **Phylogenetic variance (*V_p_*)** | **Residual variance (*e*)** |
| --- | --- | --- | --- |
| Without phage | Maximum mortality (%) | 37.52 (1.18, 98.44) | 6.04 (4.75, 7.37) |
|  | Mortality T50 (hours) | 86.64 (3.07, 232.84) | 8.62 (6.77, 10.67) |
|  | Maximum melanisation (%) | 12.17 (0.00, 36.63) | 5.68 (4.27, 6.92) |
|  | Melanisation T50 (hours) | 19.80 (4.09, 39.65) | 2.09 (1.68, 2.56) |
| With phage | Maximum mortality (%) | 122.97 (19.68, 290.39) | 7.12 (5.45, 8.73) |
|  | Mortality T50 (hours) | 97.20 (9.45, 258.79) | 8.99 (7.00, 11.02) |
|  | Maximum melanisation (%) | 29.08 (0.02, 70.89) | 4.67 (3.64, 5.91) |
|  | Melanisation T50 (hours) | 20.04 (5.89, 39.22) | 1.93 (1.55, 2.35) |

**Supplementary Figures**

**Figure S1: Correlation between the weight and pixel area of *G. mellonella* larva**. The relationship between weight and pixel area of *G. mellonella* larva assessed using least-squares linear regression (r = 0.94, p <0.001, n = 120).

**Figure S2: Pilot *G. mellonella* survival data under different inoculation conditions.** A) Survival of larvae over time when injected with varying doses of *S. aureus* 13S44S9 via septic pin prick (needle) or with a syringe. B) Survival of larvae injected with 0.5x10^8^ bacteria from a subset of tested *Staphylococcal* strains. C) Survival of larvae co-inoculated with 0.5x10^8^ *S. aureus* 13S44S9 and varying MOIs of ISP.

**Figure S3: Automated image segmentation algorithm.** Images show an example plate of inoculated *G. mellonella* larvae after the image has been demosaiced and white balanced (A), and then as a false colour validation image showing segmentation masks (B). In the false colour image, the orange area represents the rectangle of 18% neutral grey card used for white balancing, the blue areas represent the individual well masks, and the purple areas the individual larva masks.

**Figure S4: Mortality and melanisation in *G. mellonella* infection controls.** Panels show the mean mortality and melanisation percentages of 120 *Galleria mellonella* larvae from each infection control treatment.

**Figure S5: Mortality curves of *G. mellonella* larvae infected with one of 64 *Staphylococcaceae* isolates in the presence or absence of bacteriophage, ISP.** Panels show the mean percentage mortality of *Galleria mellonella* larvae (solid lines) infected with an isolate of *Staphylococcaceae* both with (blue) and without (red) the bacteriophage ISP. Lines illustrate the non-linear least squares model fits for sigmoidal mortality curves under the “cured” assumption (i.e., where the endpoint observed mortality is considered the upper asymptote of each curve).

**Figure S6: Melanisation curves of *G. mellonella* larvae infected with one of 64 *Staphylococcaceae* isolates in the presence or absence of bacteriophage, ISP.** Panels show the mean percentage melanisation of *Galleria mellonella* larvae (solid lines) infected with an isolate of *Staphylococcaceae* both with (blue) and without (red) the bacteriophage ISP. Lines illustrate the non-linear least squares model fits for Michaelis-Menten curves.

**Figure S7: Non-linear least squares model fits for mortality and melanisation data.** Plots show the relationship between model predicted (x axis) and observed (y axis) values for mortality and melanisation. Predicted values are taken from non-linear least squares models of sigmoidal curves under the “cured” assumption (mortality, left-hand plot) or Michaelis-Menten curves (right-hand plot). R^2^ values and significances of the correlation between predicted and observed values are taken from linear regression models.

**Figure S8: Inter-strain correlations between mortality and melanisation parameters in the presence of ISP.** Plots show the inter-strain correlations of each pair of mortality and melanisation parameters, with points indicating the mean value of each *Staphylococcaceae* strain. Trendlines, correlation coefficients and slopes are taken from phylogenetic mixed models of structure (1), with trendlines whose coefficient and slope estimates do not include zero highlighted in blue.

**Figure S9: Inter-strain correlations between *in vivo* and *in vitro* measures of ISP efficacy.** Plots show the inter-strain correlations of each pair of *in vivo* (maximum mortality, mortality T_50_, maximum melanisation, and melanisation T_50_) and *in vitro* (OD) measures of ISP efficacy, with points indicating the mean value of each *Staphylococcaceae* strain. Trendlines, correlation coefficients and slopes are taken from phylogenetic mixed models of structure (1). Estimates are not credible if their 95% CIs overlap zero.

**Supplementary References**

1.	Housworth EA, Martins EP, Lynch M. The Phylogenetic Mixed Model. *American Naturalist* 2004;**163**:84–96. https://doi.org/10.1086/380570

2.	Pagel M. Inferring the historical patterns of biological evolution. *Nature* 1999;**401**:877–884. https://doi.org/10.1038/44766

3.	Freckleton RP, Harvey PH, Pagel M. Phylogenetic Analysis and Comparative Data: A Test and Review of Evidence. *Am Nat* 2002;**160**:712–726. https://doi.org/10.1086/343873

4.	Hadfield JD. MCMC Methods for Multi-Response Generalized Linear Mixed Models: The MCMCglmm R Package. *J Stat Softw* 2010;**33**:1–22. https://doi.org/10.18637/jss.v033.i02

5.	Team RC. R: A language and environment for statistical computing. 2020.
